# Dynamic Thermal Properties Estimation Using Sensitivity Coefficients for Rapid Heating Process

**DOI:** 10.3390/foods10081954

**Published:** 2021-08-22

**Authors:** Anbuhkani Muniandy, Patnarin Benyathiar, Dharmendra K. Mishra, Ferhan Ozadali

**Affiliations:** 1 Department of Food Science, Purdue University, 745 Agriculture Mall Dr, West Lafayette, IN 47907, USA; amunian@purdue.edu (A.M.); fozadali@purdue.edu (F.O.); 2 Department of Food Technology, Mahidol University, Kanchanaburi Campus, 199 Sangkraburi Road, Sai Yok, Kanchanaburi 71150, Thailand; patnarin.ben@mahidol.ac.th; 3 Mead Johnson Nutrition, Reckitt Benckiser Health, 2400 W Lloyd Expy, Evansville, IN 47712, USA

**Keywords:** temperature-dependent thermal properties, scaled sensitivity coefficient, TPCell, parameter estimation, inverse problems

## Abstract

Thermal conductivity determination of food at temperatures > 100 °C still remains a challenge. The objective of this study was to determine the temperature-dependent thermal conductivity of food using rapid heating (TPCell). The experiments were designed based on scaled sensitivity coefficient (SSC), and the estimated thermal conductivity of potato puree was compared between the constant temperature heating at 121.10 °C (R12B10T1) and the rapid heating (R22B10T1). Temperature-dependent thermal conductivity models along with a constant conductivity were used for estimation. R22B10T1 experiment using the *k* model provided reliable measurements as compared to R12B10T1 with thermal conductivity values from 0.463 ± 0.011 W m^−1^ K^−1^ to 0.450 ± 0.016 W m^−1^ K^−1^ for 25–140 °C and root mean squares error (*RMSE*) of 1.441. In the R12B10T1 experiment, the analysis showed the correlation of residuals, which made the estimation less reliable. The thermal conductivity values were in the range of 0.444 ± 0.012 W m^−1^ K^−1^ to 0.510 ± 0.034 W m^−1^ K^−1^ for 20–120 °C estimated using the *k* model. Temperature-dependent models (linear and *k* models) provided a better estimate than the single parameter thermal conductivity determination with low *RMSE* for both types of experiments. SSC can provide insight in designing dynamic experiments for the determination of thermal conductivity coefficient.

## 1. Introduction

In food processing, experiments designed under dynamic heating conditions for estimation of thermal conductivity at elevated temperatures have received much attention recently due to the development and implementation of novel and innovative technologies. Given this, innovative product and process development in a very competitive market demands the development of challenging products, which will require the determination of their thermal properties under realistic processing conditions. The inverse problems technique is an effective tool which can be used to solve emerging challenges in food manufacturing [1,2,3,4,5]. Due to the lack of rapid methods, estimation of thermal properties is usually performed from experiments in a constant temperature environment [1]. The parameter estimation technique has been widely used in estimating the thermal properties of various food products [3,5,6,7,8,9,10,11,12,13,14,15]. It has also been used to estimate the fluid-to-particle heat transfer coefficient during aseptic processing of particulate foods [16] and heat flux during baking [17]. Constant temperature boundary condition can lead to prolonged exposure of heat to the sample. This can potentially degrade the product and then reliable estimates of thermal properties may not be obtained. Studies in the literature have used linear and non-linear models for the estimation of the thermal conductivity from the experimental temperature profile [1,2,18]. However, most of those studies worked on the slow heating experiments.

The objective of this study was to determine temperature-dependent thermal conductivity utilizing an experimental design based on scaled sensitivity coefficients (SSC). Parameter estimation was used to estimate constant and temperature-dependent thermal conductivity using experimental temperature profiles. The parameter SSC were studied to determine if the parameter can be estimated with relative accuracy associated with it [19,20]. A comparison of thermal conductivity estimation was presented using constant temperature boundary condition (R12B10T1, represents the traditional approach) vs. heat flux boundary condition (R22B10T1, represents the rapid heating method). The numbering systems (R12B10T1 and R22B10T1) used to describe the experiments were adopted from transient heat conduction solutions [21]. A high fat containing product to simulate soups that are high in fat content was chosen as a model food to compare the thermal properties between R12B10T1 and R22B10T1.

## 2. Materials and Methods

### 2.1. Sample Preparation

Potato puree, containing 22% (*w*/*w*) fat, was prepared with chicken broth, heavy cream, potato flakes, and butter. The ingredients in a vessel were heated on a hot plate at medium heat until reaching the temperature of 95 ± 2 °C and then the vessel was removed from the hot plate to cool down to room temperature before further analysis. The apparent viscosity of the sample was 16,735 cP measured by Brookfield AMETEK DVE Viscometer (Middleboro, MA, USA) at 6.27 s^−1^ with LV s64 spindle.

### 2.2. Mathematical Model for Transient Heat Conduction in Cylindrical Coordinate for Constant Temperature Boundary Condition (R12B10T1) Experiment

The predicted temperature profile for the R12B10T1 experiment was obtained based on the finite element numerical solution of 2D axisymmetric heat transfer equation in COMSOL (Burlington, MA, USA), as shown in Equation (1). The domain of the heat transfer included the thermocouple, sample, and stainless–steel cup. A predefined mesh size calibrated for heat transfer was used for the entire geometry with a total of 2734 elements. The minimum element size was 0.516 mm with an average of 0.915 mm. The total mesh area and element area ratio were 1783 mm^2^ and 1.423 × 10^4^, respectively.

(1) $$\frac{1}{r}\frac{\partial }{\partial r}\left[kf_{k}(T,k)r\frac{\partial T}{\partial r}\right]+\frac{\partial }{\partial z}\left[kf_{k}(T,k)\frac{\partial T}{\partial z}\right]=Cf_{c}(T,k)\frac{\partial T}{\partial t}\;for\;R_{A}<r\leq R_{B},\;0<z\leq Z_{A},\;t>0$$

The boundary conditions were,

(2) $$\frac{\partial T}{\partial r}\left(R_{A},z,t\right)=T(t),\;\frac{\partial T}{\partial z}(r,0,t)=T(t),\;\frac{\partial T}{\partial z}(r,Z_{A},t)=T(t)\;$$

The initial temperature was,

(3) $$T(r,z,0)=T_{o}\;$$

**Figure 1:**
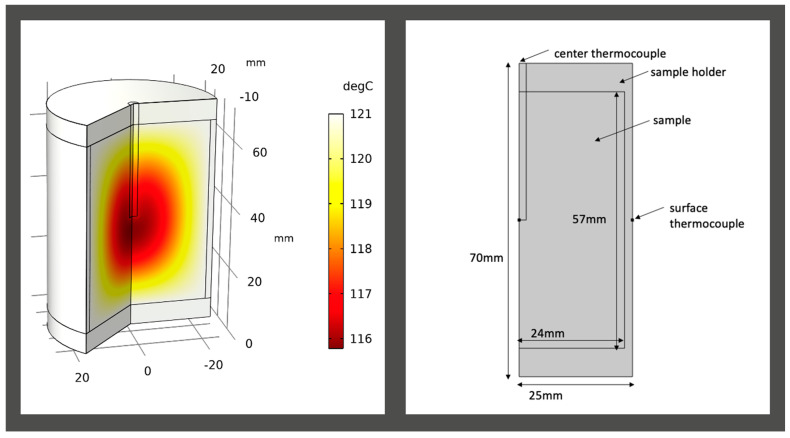
Simulation of R12B10T1 case using numerical solution. The solution shown was obtained at 40 min.

For the R12B10T1 experiment, the sample was placed in a cylindrical stainless-steel 316L sample holder, which contained a thermocouple probe at the geometric center (Figure 1). Another thermocouple was placed on the external surface of the sample holder and secured with Kapton^®^ polyimide tape (DuPont, Wilmington, DE, USA). The initial temperature $\left(T_{o}\right)$ of the sample was ~20 °C. Prior to starting the experiment, the temperatures of the sample and sample holder were equilibrated for 10 min. The sample holder was pressurized to 30 psig and placed in a silicone oil bath that was set at 121.10 °C. The center and surface thermocouples were used to monitor temperature at the center and at the surface, respectively, using LabView (National Instruments, Austin, TX, USA) as the data acquisition software. Once the sample was placed in the oil bath, the experiment was performed until the center thermocouple reached 120 °C. To terminate the experiment, the sample holder was removed from the oil bath and cooled to room temperature before releasing the pressure. Triplicate analyses were executed for statistically verifiable data.

### 2.3. Mathematical Model for Transient Heat Conduction in a Hollow Cylinder with Heat Flux on the Inside for Rapid Heating Condition (R22B10T1) Experiment

Measurement of thermal conductivity by the TPCell device is based on R22B10T1 in a hollow cylinder with a heater located at the center [2]. The equations are shown below,

(4) $$\frac{1}{r}\frac{\partial }{\partial r}\left[k_{h}r\frac{\partial T}{\partial r}\right]+\frac{\partial }{\partial z}\left[k_{h}\frac{\partial T}{\partial z}\right]+g_{0}f(t)=C_{h}\frac{\partial T}{\partial t}\;for\;R_{0}<r\leq R_{1,},\;\;0<z\leq Z_{1},\;t>0\;$$

(5) $$\frac{1}{r}\frac{\partial }{\partial r}\left[kf_{k}(T,k)r\frac{\partial T}{\partial r}\right]+\frac{\partial }{\partial z}\left[k_{1}f_{k}(T,k)\frac{\partial T}{\partial z}\right]=Cf_{C}(T,k)\frac{\partial T}{\partial t}\;for\;R_{1}<r\leq R_{2},\;0<z\leq Z_{1},\;t>0\;$$

The insulation boundary conditions were used due to the short duration of experiment [2],

(6) $$\frac{\partial T}{\partial r}\left(R_{2},z,t\right)=0,\;\frac{\partial T}{\partial z}(r,0,t)=0,\;\frac{\partial T}{\partial z}(r,Z_{1},t)=0\;$$

The initial condition was,

(7) $$T(r,z,0)=T_{0}\;$$

**Figure 2:**
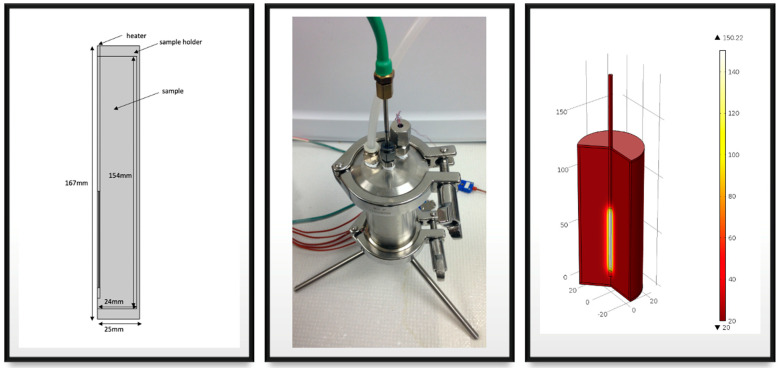
Schematic of R22B10T1 case: TPCell instrument with the center heater (**left**), and simulation of the center heater with product (**right**).

The thermal conductivity of the samples was measured using the TPCell by loading 275 mL of the sample into the cylindrical sample holder (Figure 2). The $T_{o}$ of the sample was ~20 °C. The sample holder was sealed and pressurized up to 60 psig using air to achieve an elevated temperature of the sample. The heater was supplied with 20 W power for the duration of the experiment. Once the temperature of the heater reached 137.55 ± 0.42 °C, the power supply was cut off to stop the experiment. The resistance (R) of the heater was converted to temperature using a calibration equation, T= 25.381R – 12,295. Triplicate analyses were performed for statistical accuracy.

### 2.4. Parameter Estimation

Thermal conductivity was estimated using a sequential estimation method from the temperature profiles of R12B10T1 and R22B10T1 experiments. The functions used for thermal conductivity estimation for both experiments were single parameter, linear, and *k* model as shown below. The *k* model was a reparameterization of the linear model to improve the parameter identifiability. 

(A) Single parameter model,

(8) $$k=k_{C}\;$$

(B) Linear model with two parameters,

(9) k(T)=a+b(T) 

(C) *k* model with two parameters,

(10) $$k(T)=k_{1}\left(\frac{T_{2}-T}{T_{2}-T_{1}}\right)+k_{2}\left(\frac{T-T_{1}}{T_{2}-T_{1}}\right)$$

### 2.5. Scaled Sensitivity Coefficient and Sequential Estimation

Parameter identifiability was assessed by plotting the SSC to determine whether all the parameters in a model can be estimated uniquely and simultaneously with their relative errors. The SSC is also used in the optimal experimental design criteria where it maximizes the determinant of the sensitivity matrix. However, in this study, SSC was used to gain further understanding with regards to parameter correlation and identifiability. The sensitivity coefficient of thermal conductivity was derived by taking the first derivative of the temperature with respect to thermal conductivity. To perform a direct comparison, the sensitivity coefficient was scaled by multiplying with the parameter to obtain the SSC as shown in Equation (11).

(11) $$X_{i}^{\prime }=k_{i}\frac{\partial T}{\partial k_{i}}$$

The sum of SSC was calculated using Equation (12). All parameters in the model cannot be estimated uniquely and simultaneously if the sum of SSC is equal to zero [19,22].

(12) $$k_{1}{\frac{\partial T}{\partial k_{1}}|}_{k_{2}}+k_{2}{\frac{\partial T}{\partial k_{2}}|}_{k1}=-(T-T_{0})-\left[C_{1}{\frac{\partial T}{\partial C_{1}}|}_{C_{2}}+C_{2}{\frac{\partial T}{\partial C_{2}}|}_{C1}\right]\;$$

Based on the experimental temperature profile, the thermal conductivity was determined using sequential estimation. The sequential estimation procedure was developed in MATLAB^®^ [19] based on the Gauss minimization method and required prior information of parameters. In this estimation procedure, the parameter estimates initially would have large fluctuations, but the estimates eventually attain a constant value once enough data have been added.

The mathematical form of non-linear sequential estimation is derived from maximum a posteriori (MAP) estimation. The minimization function in the Gauss method can be expressed as;

(13) $$S={\left[Y-\overset{\wedge }{Y}(\beta )\right]}^{\prime }W\left[Y-\overset{\wedge }{Y}(\beta )\right]+{\left[\mu -\beta \right]}^{\prime }U\left[\mu -\beta \right]\;$$

where Y is the experimental response variable and $\hat{Y}$ is the predicted response, μ is the prior information of parameter vector β, W is the inverse of covariance matrix of errors, and U is the inverse covariance matrix of parameters. β was solved and reported as the estimated thermal conductivity. The parameter estimates were reported along with its root mean square error (*RMSE*) and 95% confidence interval. The *RMSE* for the estimate was calculated based on Equation (14). The 95% confidence interval of parameter were calculated using MATLAB^®^ built-in function nlparci (parameter, residual, sensitivity coefficient). Residuals were calculated by taking the difference between the experimental and predicted temperature at each time point. Standard statistical assumptions of uncorrelated errors, which are normally distributed with zero mean and constant variance, were verified for the residuals. Additional assumptions specific to the use of sequential estimation, which needs to be satisfied, are known as covariance matrix errors, no errors in independent variables, and the known prior of information of parameters.

(14) $$RMSE=\sqrt{\frac{\sum_{i=1}^{n}{\left(\hat{Y_{i}}-Y_{i}\right)}^{2}}{n}}$$

## 3. Results and Discussion

**Figure 3:**
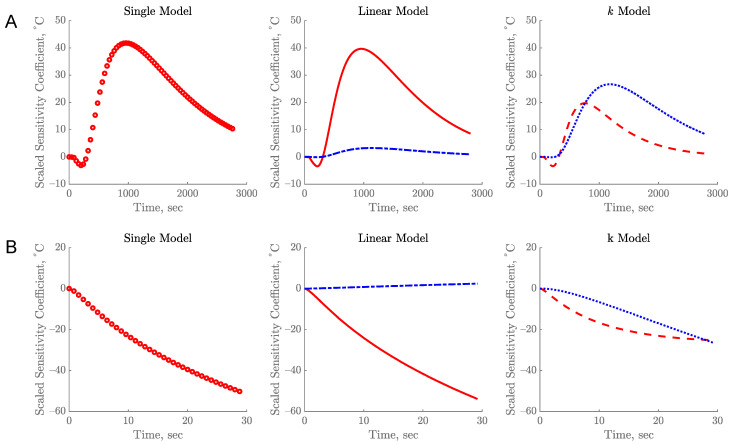
Scaled Sensitivity Coefficient (SSC) of thermal conductivity for single parameter model, linear model, and *k* model for R12B10T1 (**A**) and R22B10T1 (**B**) experiments. Legends: (o) *k_c_*, (–) a, (–.) b, (– –) *k*_1_, and (…) *k*_2_.

Parameters of Equations (8–10) showed large SSC as illustrated in Figure 3. The plots were used to determine if the simultaneous estimation of parameters was possible. All parameters in the model can be estimated with a low error when the magnitude of the SSC is large and without linear dependency or correlation between the parameters [19]. To consider SSC to be large, it should be at least 10% of the temperature rise [1]. When SSC is small, the estimation may result in larger errors and hence larger confidence intervals of the parameter. Parameters are considered not correlated if their ratio was not constant [20]. Visually, the SSC curves would have the same pattern with the same or different magnitudes if the parameters are correlated. 

For the R12B10T1 experiment, the SSC plots of the parameters for estimation of thermal conductivity were large and uncorrelated, as shown in Figure 3A. Based on the result from the single parameter, SSC was 41.68%, which is considered large. The SSCs of two parameters for estimation of thermal conductivity using the linear model were 39.65% and 3.17%, as compared to 15.78% and 22.18% for the *k* model and the sums of SSC for those models were not zero. The two parameters estimated using the linear and *k* models were not correlated as a result. This means both equations can be used to estimate the thermal conductivity with two parameters using the inverse problems methods. However, the SSC of parameter *b* in linear model was very small (3.17%) as compared to the parameter *a* (~39.65%), suggesting that it would be difficult to estimate *b* and probably would have large standard error. The magnitude SSC of both parameters *k*_1_ and *k*_2_ in the *k* model are evenly distributed as compared to the temperature rise. The SSC plots for the two parameters were quite identical to the plots reported previously [1]. This can be attributed to the identical nature of the R12B10T1 experiment and measurement of temperature at the geometric center of a cylindrical container.

Figure 3B shows the SSC plots of the parameters for estimation of thermal conductivity from the R22B10T1 experiment. The SSC for a single parameter was 44.6%. The values of SSCs for the two parameters estimated using the linear model were 53.90% and 2.40%, as compared to 21.25% and 22.08% for the *k* model. The parameter *b* in the linear model had the lowest SSC and hence it would be harder to estimate. The SSC of parameters estimated from this experiment also exhibited a large magnitude with no correlation between parameters. The sum of SSC for all parameters was not zero, which means the parameters can be estimated uniquely and simultaneously. In both experiments, the magnitude of the SSC was reduced in the linear and the *k* model when an additional parameter was added (Figure 3). This is because the magnitude of SSC with one parameter is now being shared by two parameters non-proportionally.

**Figure 4:**
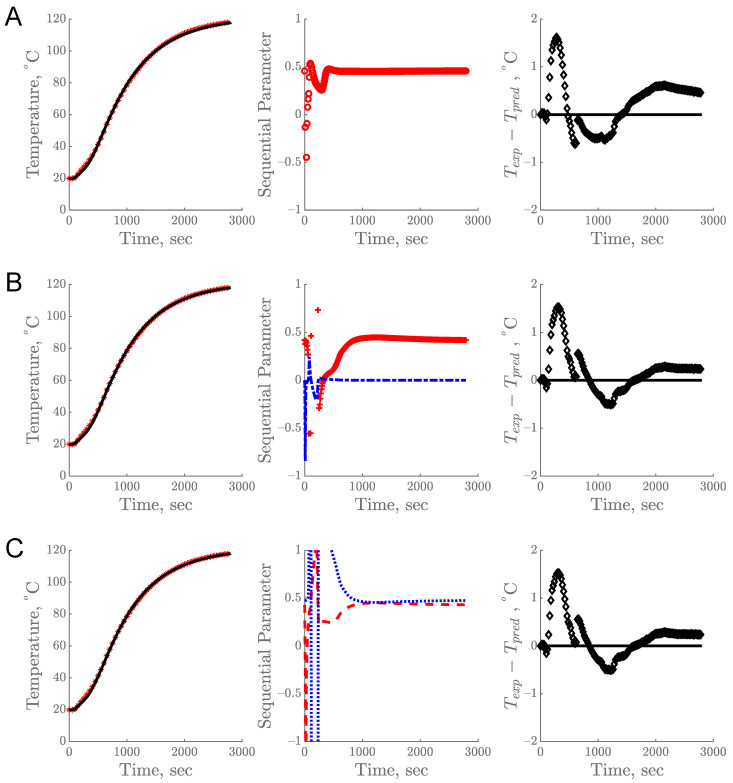
Experimental vs. predicted temperature profile over time (**left**), sequential estimation of parameters (**center**), and corresponding residual plot of experimental (T_exp_) and predicted (T_pred_) temperature (**right**) for R12B10T1 experiment: (**A**) single parameter model, (**B**) linear model, and (**C**) *k* model. Legends: (*) T_exp_, (–) T_pred_, (o) *k_c_*, (+) a, (–.) b, (– –) *k*_1_, (…) *k*_2,_ and (◊) residuals.

In order to estimate the parameters with low errors, the R12B10T1 experiment must be conducted for at least 20 min while the R22B10T1 experiment required only 30 s (Figure 3). The experimental time is optimal when the parameter SSC attains a maximum value. The optimal experimental time is further confirmed when a constant value is achieved by the sequential estimation (Figure 4). Further data acquisition beyond this optimal time may not add any substantial improvement to the estimated parameter [20]. The large difference in the experiment duration was due to the boundary conditions used in these experiments. The R12B10T1 experiment had constant temperature boundary on the walls of the sample holder, hence the temperature rise at the geometric center was relatively slow which led to longer experiment duration. In contrast, the R22B10T1 experiment utilizes a heat flux boundary at the center of the sample resulting in rapid temperature rise. The SSC indicates the magnitude of change in the temperature due to perturbation in the parameter [19]. Due to the different boundary condition used, the duration to reach the highest magnitude of SSC was different between these two experiments.

The sequential estimation of parameters based on the temperature profile obtained from the R12B10T1 experiment is shown in Figure 4. The predicted data from all three models showed a good fit with the experimental data. This estimation process requires appropriate prior information as the initial guess. During sequential estimation, the estimated parameter values keep changing as each datum is being added, with the goal of minimizing the sum of squares of the errors as illustrated in Figure 4A–C (center). The estimation was complete and reliable when parameter values attained a constant value and remained constant for the rest of the experimental time. When the parameter values do not attain a constant value toward the end of the experiment, it indicates that there might be some error in the model or in the experiment [2]. The final estimated values were reported along with their standard error and 95% confidence interval (Table 1).

The residuals from all models in Figure 4 show a pattern, which is not desirable, and the mean value of the residuals was 0.27 for the constant model and 0.17 for both the linear and *k* model. These residuals were most likely due to a potential change in the sample during prolonged heating. A similar result was also reported from retort processing of cherry pomace [1]. Prolonged exposure of heat to a food product at a high temperature can lead to undesirable reactions within the food matrix including oxidation, separation of lipid and moisture, the formation of unwanted off-aroma/off-flavor compounds, browning, and degradation of nutrient and sensory quality attributes.

Overall, the estimation from R12B10T1 experiment had relatively low *RMSE* as shown in Table 1. The highest *RMSE* was observed for the single parameter model in all three replicates while the lowest *RMSE* was found for the linear and *k* model. Due to the parameter uncertainty with large relative error and large confidence interval of *b* in the linear model, the *k* model was chosen as the best thermal conductivity model.

**Figure 5:**
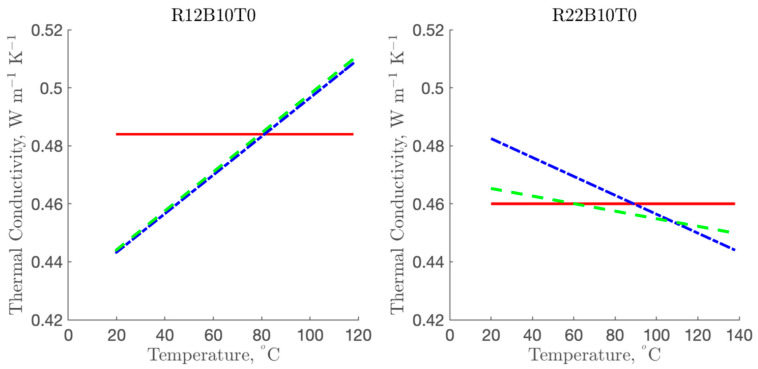
Thermal conductivity of potato puree estimated using the single parameter model, linear model, and *k* model for R12B10T1 and R22B10T1 experiments. Legends: (–) single parameter model, (–.) linear model, (– –) *k* model.

For the single parameter model of R12B10T1, the thermal conductivity value of potato puree was constant at 0.484 ± 0.024 W m^−1^ K^−1^ as shown in Figure 5. An increase in thermal conductivity was observed in both the linear and *k* models. The *k* model showed the thermal conductivity values from 0.444 ± 0.012 W m^−1^ K^−1^ to 0.510 ± 0.034 W m^−1^ K^−1^ while those from the linear model were 0.447 ± 0.013 W m^−1^ K^−1^ to 0.523 ± 0.038 W m^−1^ K^−1^. Thermal conductivity value of mashed potato in literature has been reported as 0.59 W m^−1^ K^−1^ [23] and blanched potato as 0.55 W m^−1^ K^−1^ at 20 °C [24]. Values reported in this study were slightly lower due to presence of high fat content which is known to decrease the thermal conductivity of foods [25,26]. The thermal conductivity value calculated from Choi-Okos model [27] based on the composition of the potato puree was 0.462 W m^−1^ K^−1^ at 25 °C which is well within the range reported in Figure 5. 

**Figure 6:**
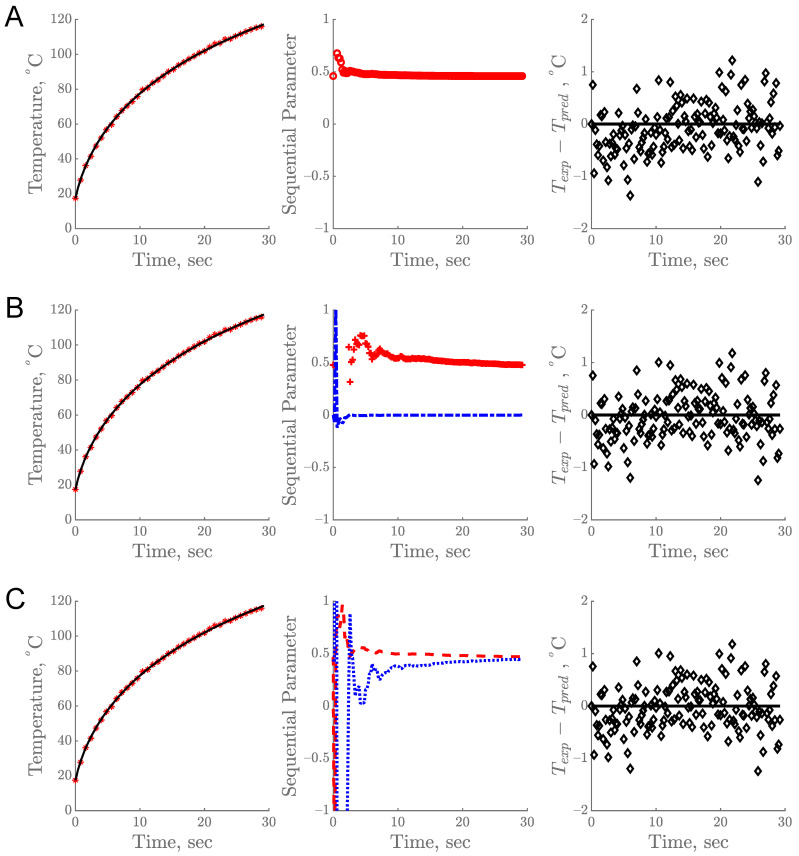
Experimental vs. predicted temperature profile over time (**left**), sequential estimation of parameters $k_{1}$ and $k_{2}$ (**center**) and corresponding residual plot (**right**) for R22B10T1 experiment: (**A**) single parameter model, (**B**) linear model, and (**C**) *k* model. Legends: (*) T_exp_, (–) T_pred_, (o) *k_c_*, (+) a, (–.) b, (– –) *k*_1_, (…) *k*_2,_ and (◊) residuals.

The sequential estimation of parameters based on the temperature profile obtained from the R22B10T1 experiment is shown in Figure 6. Based on the result, the model predicted temperature fits well with the experimental data. The sequential estimation from this experiment showed that parameter values remain unchanged toward the end of the experiment. The residuals for all models did not violate any standard statistical assumption. The average of residuals for linear and *k* model was −0.05. The mean value of the residuals from the R22B10T1 experiment were much smaller compared to the residuals in R12B10T1. This confirms that the parameter estimation from the R22B10T1 experiment was reliable. In this case, the R22B10T1 experiment was only 30 s as compared to 47 min for the R12B10T1 experiment. The parameter covariance matrix and correlation matrix for the linear and *k* model are presented in Table 2. The correlation coefficient of parameters in the linear model was quite high (0.99), which is not desirable when estimating multiple parameters. This was expected based on the SSC of parameters *a* and *b*.

The RMSEs from R22B10T1 were higher than those from R12B10T1 due to the differences in accuracy of the temperature sensing elements. Based on the results from Table 1, the least *RMSE* values from the R22B10T1 were observed for the linear and *k* models. The *RMSE* from R22B10T1 of the linear and *k* models were close, which was not seen in the R12B10T1 experiment. Since the parameter *b* for the linear model exhibited large relative error and confidence interval (Table 1), it is not considered as the right model for the conductivity. Generally, thermal conductivity changes with temperature. Thus, the single parameter model is an average value over the temperature range [27]. Although it can be used for initial assessment, the *k* model would be appropriate and realistic.

Based on the results in Figure 5, the thermal conductivity result of potato puree using R22B10T1 showed that the values of the single parameter model remained constant over the temperatures at 0.460 ± 0.003 W m^−1^ K^−1^ while the average values of the linear model and *k* model decreased from 0.481 ± 0.010 W m^−1^ K^−1^ to 0.444 ± 0.010 W m^−1^ K^−1^ and 0.463 ± 0.011 W m^−1^ K^−1^ to 0.450 ± 0.016 W m^−1^ K^−1^, respectively. The major difference using the R22B10T1 and R12B10T1 experiments was the variation of thermal conductivity with the increase in temperatures. The thermal conductivity values obtained from R22B10T1 showed a decrease in values with increasing temperature while an increasing trend was observed from the R12B10T1 experiment. While the thermal conductivity is known to increase with temperature, a slight decrease is evident in foods with high fat content [27]. Up to 20.4% decrease in thermal conductivity can occur in pure fat at temperatures between 25 °C to 140 °C [28]. In the current study, the decrease in thermal conductivity over the same temperature range was 8.3% and 2.8% for the linear and *k* model, respectively.

The temperature abuse during the experiment could negatively impact the reliability of estimated thermal properties. The separation of potato puree and potential changes in its matrix could occur due to prolonged exposure to the high temperature. This degradation phenomenon was observed from the R12B10T1 experiment. The increasing trend in the thermal conductivity values obtained from the R12B10T1 experiment might have an error due to the changes in the food matrix.

Even though the inverse problems were able to estimate the parameters of the linear model, the relative error and the confidence intervals were large for the parameter *b*. In addition, the correlation coefficient of parameters of the linear model was higher (~0.99) as compared to the *k* model (~0.95). A study by da Silva et al. (2020) reported that simultaneous estimation of two parameters for determination of thermal diffusivity of coconut pulp was not possible due to the high correlation between the parameters, this is similar to what was observed in the current study with the linear model. The *k* model can be seen as a reparameterization of the linear model and had the same *RMSE* (Table 1). Reparameterization of the linear model to the *k* model improved the relative error, tightened the band width of the confidence intervals, and decreased the correlation between the parameters (Table 1 and Table 2). Based on the SSC, sequential estimation, residual analysis, correlation coefficient, and *RMSE* values, the *k* function could be an appropriate model of temperature-dependent thermal conductivity for high fat-containing purees.

## 4. Conclusions

Innovations in the food industry toward rapid heating technologies such as ohmic and microwave heating requires thermal properties that are determined in realistic experimental conditions. Thermal property determination using rapid heating is suitable for novel applications in the food industry. The thermal conductivity of food was determined for both R12B10T1 (constant temperature boundary conditions) and R22B10T1 (heat flux boundary conditions) dynamic experiments using SSC and sequential estimation. The *k* model was sufficient in describing the dependence of thermal conductivity with temperature for both experiments. The linear model showed a large confidence interval of estimated parameters and high correlation between parameters. The thermal abuse created by the R12B10T1 experiment might have caused the higher conductivity measurements in both the single parameter model and temperature-dependent models due to the prolonged exposure at elevated temperatures. The new approach of rapid heating with TPCell, therefore, provides a quick and realistic measurement of the thermal conductivity in the food processing temperature range of 20–140 °C. This study will be beneficial to the food industry as a user-friendly tool for measuring thermal properties at elevated temperatures.

## Figures and Tables

**Table 1:** Estimation of the thermal conductivity in the single parameter model, linear model, and *k* model for R12B10T1 and R22B10T1 experiments.

Model		R12B10T1			R22B10T1		
		Rep 1	Rep 2	Rep 3	Rep 1	Rep 2	Rep 3
Single parameter model	*k_c_*	0.457	0.490	0.504	0.464	0.459	0.458
	*RMSE*	0.582	0.663	0.838	1.400	1.540	1.485
	$LCI_{k_{C}}$	0.456	0.490	0.503	0.462	0.458	0.457
	$UCI_{k_{C}}$	0.457	0.491	0.505	0.465	0.460	0.459
Linear model	*a*	0.421	0.435	0.435	0.505	0.478	0.485
	*b ×* 10^−3^	0.438	0.685	0.873	−0.515	−0.239	−0.224
	*RMSE*	0.485	0.436	0.525	1.285	1.538	2.275
	*RE_a_* %	0.174	0.165	0.157	1.179	0.915	0.783
	*RE_b_* %	2.008	1.292	0.985	−14.331	−22.417	−21.015
	*LCI_a_*	0.419	0.433	0.433	0.491	0.467	0.471
	*UCI_a_*	0.423	0.437	0.437	0.519	0.489	0.499
	*LCI_b_* × 10^−3^	0.415	0.663	0.847	−0.692	−0.379	−0.394
	*UCI_b_* × 10^−3^	0.463	0.706	0.898	−0.338	−0.099	−0.053
*k* model	*k*_1_	0.430	0.449	0.452	0.484	0.469	0.450
	*k*_2_	0.474	0.517	0.540	0.437	0.445	0.468
	*RMSE*	0.485	0.436	0.525	1.285	1.538	1.499
	*RE_k_*_1_ %	0.129	0.122	0.114	0.616	0.508	0.495
	*RE_k_*_2_ %	0.075	0.073	0.071	0.889	0.679	0.603
	$LCI_{k_{1}}$	0.428	0.447	0.451	0.477	0.463	0.444
	$UCI_{k_{1}}$	0.431	0.450	0.454	0.491	0.475	0.456
	$LCI_{k_{2}}$	0.473	0.516	0.538	0.427	0.437	0.461
	$UCI_{k_{2}}$	0.475	0.518	0.541	0.446	0.453	0.475

**Table 2:** Covariance and correlation matrices for the linear and *k* model for R12B10T1 and R22B10T1 experiments.

			Rep 1		Rep 2		Rep 3	
**R12B10T2**			***a***	***b***	***a***	***b***	***a***	***b***
Linear model	Covariance × 10^−7^	*a*	10.258	−0.122	8.014	−0.097	10.512	−0.130
		*b*	−0.122	0.002	−0.097	0.001	−0.130	0.002
	Correlation	*a*	1.000	−0.988	1.000	−0.985	1.000	−0.983
		*b*	−0.988	1.000	−0.985	1.000	−0.983	1.000
			*k*_1_	*k*_2_	*k*_1_	*k*_2_	*k*_1_	*k*_2_
*k* model	Covariance × 10^−7^	*k*_1_	5.964	−3.288	4.612	−2.686	5.979	−3.698
		*k*_2_	−3.288	2.412	−2.686	2.189	−3.698	3.279
	Correlation	*k*_1_	1.000	−0.867	1.000	−0.845	1.000	−0.835
		*k*_2_	−0.867	1.000	−0.845	1.000	−0.835	1.000
**R22B10T2**			***a***	***b***	***a***	***b***	***a***	***b***
Linear model	Covariance × 10^−5^	*a*	4.782	−0.059	3.697	−0.045	6.105	−0.075
		*b*	−0.059	0.001	−0.045	0.001	−0.075	0.001
	Correlation	*a*	1.000	−0.996	1.000	−0.996	1.000	−0.996
		*b*	−0.996	1.000	−0.996	1.000	−0.996	1.000
			*k*_1_	*k*_2_	*k*_1_	*k*_2_	*k*_1_	*k*_2_
*k* model	Covariance × 10^−5^	*k*_1_	1.200	−1.493	1.096	−1.329	0.911	−1.102
		*k*_2_	−1.493	2.037	−1.329	1.769	−1.102	1.464
	Correlation	*k*_1_	1.000	−0.955	1.000	−0.955	1.000	−0.955
		*k*_2_	−0.955	1.000	−0.955	1.000	−0.955	1.000

## Nomenclature

R12B10T1	transient heat conduction in cylindrical coordinate for constant temperature boundary condition
R22B10T1	transient heat conduction in a hollow cylinder with heat flux on the inside and insulated on the outside for rapid heating condition
*RMSE*	root meat square error
*a*, *b*	parameters in linear model
β	parameter
$X_{i}^{\prime }$	scaled sensitivity coefficient, °C
μ	prior information of parameter vector, β, W m^−1^ K^−1^
$C_{h}$	volumetric capacity of heater, J m^−3^ K^−1^
$C_{TC}$	volumetric capacity of thermocouple, J m^−3^ K^−1^
$C_{1}$	volumetric heat capacity of sample, J m^−3^ K^−1^ at $\;T_{1}$
$C_{2}$	volumetric heat capacity of sample, J m^−3^ K^−1^ at $T_{2}$
$f_{k}$	dimensionless thermal conductivity as a function of temperature
$f_{C}$	dimensionless heat capacity as a function of temperature
$g_{0}$	power, W m^−3^
k	thermal conductivity of sample, W m^−1^ K^−1^
$k_{h}$	thermal conductivity of heater, W m^−1^ K^−1^
$k_{TC}$	thermal conductivity of thermocouple, W m^−1^ K^−1^
$k_{C}$	thermal conductivity in single parameter model, W m^−1^ K^−1^
$k_{1},\;k_{2}$	parameters used in *k* model
LCI	95% lower confidence interval of parameters
i	index
n	number of responses
r	radial position, m
R	resistance of the heater, Ω
$R_{0}$	centerline of the cylinder, $R_{0}=0$
$R_{1}$	interface of heater and sample, m
$R_{2}$	wall of the cup, m
$R_{A}$	interface of thermocouple and sample, m
$R_{B}$	wall of the cup, m
*RE*	Relative error of parameter
T	temperature, °C
T(t)	temperature at t, °C
$T_{0}$	initial temperature of sample, °C
$T_{1}$	initial temperature for thermal properties, °C
$T_{2}$	final temperature for thermal properties, °C
t	time, s
U	inverse covariance matrix of parameters
UCI	95% upper confidence interval of parameters
W	inverse of covariance matrix of errors
Y	experimental response variable
$\hat{Y}$	predicted response variable
*Z_A_*	total height of cylinder for (R12B10T1) experiment, mm
*Z*_1_	total height of cylinder for (R22B10T1) experiment, mm
z	axial position, m
